# OsRap2.6 transcription factor contributes to rice innate immunity through its interaction with Receptor for Activated Kinase-C 1 (RACK1)

**DOI:** 10.1186/1939-8433-5-35

**Published:** 2012-12-11

**Authors:** Mwathi Jane Wamaitha, Risa Yamamoto, Hann Ling Wong, Tsutomu Kawasaki, Yoji Kawano, Ko Shimamoto

**Affiliations:** Laboratory of Plant Molecular Genetics, Nara Institute of Science and Technology, 8916-5 Takayama, Ikoma, Nara, 630-0192 Japan; Universiti Tunku Abdul Rahman Jalan Universiti, Bandar Barat, Kampar 31900 Malaysia; Department of Advanced Bioscience, Graduate School of Agriculture, Kinki University, 3327-204 Nakamachi, Nara, 631-8505 Japan

**Keywords:** OsRac1, OsRap2.6, RACK1, OsMAPK3/6, BiFC

## Abstract

**Background:**

The rice small GTPase OsRac1 is a molecular switch in rice innate immunity. The Receptor for Activated Kinase C-1 (RACK1) interacts with OsRac1 to suppress the growth of the rice blast fungus, *Magnaporthe oryzae*. RACK1 has two homologs in rice, RACK1A and RACK1B. Overexpressing RACK1A enhances resistance to the rice blast fungus. However, RACK1A downstream signals are largely unknown.

**Results:**

Here, we report the identification of OsRap2.6, a transcription factor that interacts with RACK1A. We found a 94% similarity between the OsRap2.6 AP2 domain and Arabidopsis Rap2.6 (AtRap2.6). Bimolecular fluorescence complementation (BiFC) assays in rice protoplasts using tagged OsRap2.6 and RACK1A with the C-terminal and N-terminal fragments of Venus (Vc/Vn) indicated that OsRap2.6 and RACK1A interacted and localized in the nucleus and the cytoplasm. Moreover, OsRap2.6 and OsMAPK3/6 interacted in the nucleus and the cytoplasm. Expression of defense genes *PAL1* and *PBZ1* as well as *OsRap2.6* was induced after chitin treatment. Disease resistance analysis using *OsRap2.6* RNAi and overexpressing (Ox) plants infected with the rice blast fungus indicated that *OsRap2.6* RNAi plants were highly susceptible, whereas *OsRap2.6* Ox plants had an increased resistance to the compatible blast fungus.

**Conclusions:**

OsRap2.6 contributes to rice innate immunity through its interaction with RACK1A in compatible interactions.

**Electronic supplementary material:**

The online version of this article (doi:10.1186/1939-8433-5-35) contains supplementary material, which is available to authorized users.

## Background

Rice production is constrained by various diseases, the rice blast fungus, *Magnaporthe oryzae* being among the most prominent (Ribot et al 2008; Couch et al 2005; Valent and Chumley 1991). This fungus accounts for major losses in crops and grain yields (Wilson and Talbot 2009). *M. oryzae* produces asexual spores that are dispersed rapidly by wind or by other means. Breeding for resistance is one of the safest ways to counteract *M. oryzae*; however, understanding the resistance mechanisms for blast fungus is still a challenge (Ribot et al 2008; Valent and Chumley 1991).

Pathogen associated molecular pattern (PAMP) triggered immunity (PTI) and effector triggered immunity (ETI) responses occur to evade pathogens such as the blast fungus (He et al 2007; Bent and Mackey 2007; Chisholm et al 2006; Jones et al 2006). PTI is the first line of defense, which requires membrane receptor proteins known as pattern recognition receptors stimulated by chitin, flagellin or elicitors. ETI is the second line of defense that requires intracellular receptors of pathogen virulence molecules called effectors, whose recognition induces ETI and is triggered by resistance (R) proteins (Kawano et al 2010; Zipfel 2008; Dangl and Jones 2001).

The PTI response occurs in seconds to minutes, leading to calcium ion fluxes and oxidative bursts, whereas structural responses such as callose deposition may take hours to days (Boudsocq et al 2010, Boller and Felix 2009). These secondary responses could result in hypersensitive responses (HR) (Nimchuk et al 2003), nitric oxide (NO) (Heath 2000) and reactive oxygen species (ROS) production (Mittler et al 1999; Jabs et al 1996). The mechanisms that link PTI to downstream signals are, however, unclear.

The OsRac1 small GTPase is a molecular switch in rice innate immunity (Ono et al 2001; Kawasaki et al 1999). Endogenous GTPase activity of OsRac1 hydrolyzes active guanosine triphosphate (GTP) to inactive guanosine diphosphate (GDP). On the other hand, guanine nucleotide exchange factors (GEFs) catalyze the exchange of inactive GDP to active GTP. The GEFs act as positive regulators leading to activation of downstream signals and, ultimately, resistance to pathogens (Paduch et al. 2001).

OsRac1 interacts with Pit through the nucleotide-binding (NB-ARC) (ARC: APAF-1, certain R gene products and CED-4) domain and is involved in the ETI response (Kawano et al 2010). The components in rice involved in PTI and ETI form part of the “defensome network” and include mitogen-activated protein kinase 3 and 6 (OsMAPK 3/6), nicotinamide adenine dinucleotide phosphate (NADPH) oxidase and co-chaperones RAR1, SGT1 and heat shock proteins 90 and 70 (Hsp90 and Hsp70) among others (Kim et al 2012; Chen et al 2010a; Kawano et al 2010; Nakashima et al 2008; Shirasu 2009Thao et al 2007; Wong et al 2007; Kawasaki et al 2006; Lieberherr et al 2005; Ono et al 2001).

To understand the role of OsRac1, transgenic rice plants expressing constitutively active (CA) (GTP-bound) or dominant negative (DN) (GDP-bound) OsRac1s were infected with the compatible (race 007) and incompatible rice blast fungus (race 031). The CA mutant had increased resistance and ROS production in when infected with the compatible race, whereas the DN mutant suppressed resistance and reduced ROS production when infected with the incompatible race (Chen et al 2010b; Ono et al 2001). These observations show the importance of OsRac1 as a signal transducer in rice and a positive regulator of disease resistance (Chen et al 2010b; Kawano et al 2010; Berken 2006; Suharsono et al 2002; Ono et al 2001; Kawasaki et al 1999).

Receptor for Activated Kinase C-1 (RACK1) was identified as a downstream target of OsRac1 (Nakashima et al 2008). RACK1, a 36-kDa protein is similar to the G-protein ß-subunit highly conserved in diverse species including plants (Adams et al 2011; McCahill et al 2002; Sondek and Siderovski 2001; Kwak et al 1997). RACK1 serves as a scaffold protein and binds phosphatases and transcription factors as well as membrane receptors (Adams et al 2011; Chen et al 2010a; Chen et al 2006). Rice has two RACK1 homologs annotated as RACK1A and RACK1B (Nakashima et al 2008). Constitutively active-OsRac1 interacts more strongly with RACK1A than the dominant negative-OsRac1. RACK1A over-expressing rice enhances the resistance to the compatible race of rice blast fungus (007) (Nakashima et al 2008). In rice, RACK1A interacts directly with OsRac1 and co-chaperones RAR1 and SGT1 and indirectly with Hsp90 and Hsp70 (Thao et al 2007). Hsp90 also specifically interacts with SGT1 (Takahashi et al 2003).

In this work, we identified a protein, OsRap2.6 that interacted with RACK1A in yeast two-hybrid assays. We also demonstrated that OsRap2.6 interacts with RACK1A and OsMAPK3/6 in the nucleus and the cytoplasm, the same place they localized. Expression of the defense genes *Phenylalanine ammonia lyase 1* (*PAL1)* and *Probenazole-inducible gene 1* (*PBZ1)* as well as *OsRap2.6* was induced in suspension cells treated with chitin. *OsRap2.6* RNAi plants had high susceptibility, whereas *OsRap2.6* overexpressing (Ox) plants had increased resistance to the compatible race (007) of rice blast fungus. However, no significant differences were found in *OsRap2.6* RNAi or Ox plants when challenged by the incompatible race (031). These results demonstrate that OsRap2.6 contributes to resistance towards the compatible race (007) of rice blast fungus.

## Results and discussion

### RACK1A interacts specifically with OsRap2.6 in yeast two-hybrid assays

**Figure 1:**
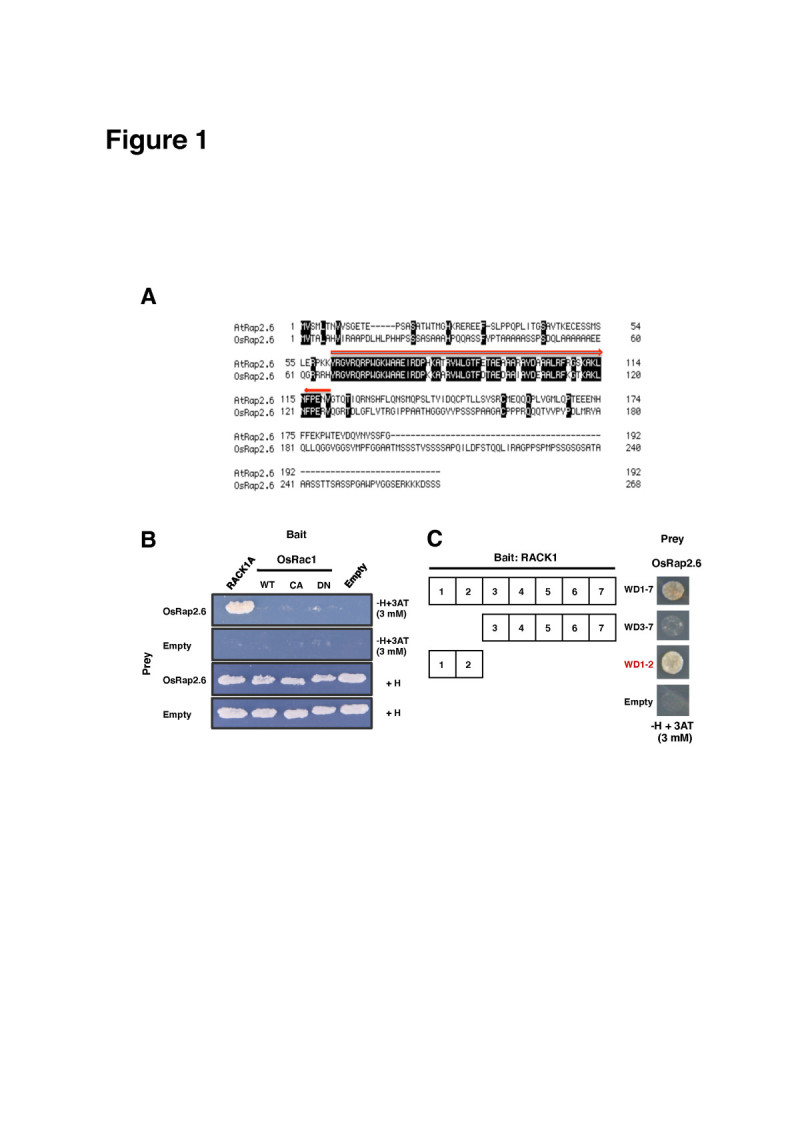
**OsRap2.6 AP2/ERF domain resembles Arabidopsis Rap2.6 and interacts with RACK1A.** (**A**) Comparison of amino acids sequences of rice and Arabidopsis Rap2.6. (**B**) Interaction of OsRap2.6 with RACK1A in yeast two-hybrid assays. OsRac1 (WT), constitutively active (CA) and dominant negative (DN)-OsRac1 mutants were examined. (**C**) Interaction of RACK1 with OsRap2.6 at WD repeat 1–2 in yeast two-hybrid assays.

Proteins that interacted with RACK1A in the rice cDNA library were screened in yeast two-hybrid (Y2H) assays. The primary candidate gene (Os04g0398000 or AK101501) had an AP2/ERF domain whose sequence shared 94% amino acid identity with Arabidopsis Rap2.6 (AtRap2.6) (shadowed regions in Figure 1A). We, therefore, named it as *Oryza sativa* Rap2.6 (OsRap2.6) and selected it for further analysis. The other candidate genes included hypothetical proteins with a MATH domain (Os01g0775300), a CaMKII association domain (Os01g0753200), or a ToIA/TF11B domain (Os12g0112600); Universal stress protein (USP) (Os5g0453700) containing a USP domain; and a V1P1-like protein whose domain was unknown (Os01g0698000) (Table 1).

**Table 1:** **RACK1A interacting proteins**

Protein name	Clone identity	Number of clones	Domain
OsRap2.6	Os04g0398000	1	AP2 domain
Hypothetical protein	Os01g0775300	1	MATH domain
Hypothetical protein	Os01g0753200	4	CaMKII association domain
Hypothetical protein	Os12g0112600	1	ToIA/TF11B domain
Universal stress protein	Os5g0453700	17	USP domain
V1P1 like protein	Os01g0698000	1	-

Proteins that interact with RACK1A identified in yeast two-hybrid assays.

The bait constructs RACK1A, OsRac1 (WT) and (CA and DN) were fused with the pBTM116ss vector. The OsRap2.6 coding region was ligated into the pVP16 prey vector. The negative controls were pBTM116ss and pVP16-Empty. The paired plasmids were transformed into the yeast *Saccharomyces cerevisiae* (L40). Positive transformants were selected based on the ability to activate transcription of the *histidine 3* (HIS3) reporter gene. We found a strong interaction between RACK1A and OsRap2.6; however, there was no observed interaction in the (WT) or the (CA and DN) OsRac1 mutants. There was no growth of colonies in the negative controls, pBTM116ss and pVP16 as expected (Figure 1B). These results demonstrated that OsRap2.6 interacts specifically with RACK1A in Y2H assays. We, therefore, hypothesised that OsRap2.6 may be functionally similar to AtRap2.6 or most members in the AP2/ERF family.

Rap2.6 is a single copy gene in the Arabidopsis genome with one AP2 domain located at the N-terminus (Nakano et al 2006). This domain has about 60 amino acids and is useful for binding DNA sequences (Magnani et al 2004). AP2/ERFs bind DNA sequences with *cis* elements such as the GCC box (AGCCGCC) and CE1 that regulates plant-pathogen interactions (Ohme-Takagi and Shinshi et al 1995). In general, AP2/ERFs are the most diverse transcription factors in plants (Riechmann and Ratcliffe 2000Ohme-Takagi and Shinshi et al. Ohme-Takagi and Shinshi 1995). AP2/ERF transcription factors are important in plant responses to abiotic and biotic stresses (Agrawal et al 2006). Arabidopsis has 145 members including Rap2.6 (Sharoni et al 2011; Sakuma et al 2002, Riechmann and Ratcliffe 2000) that confers resistance to *Pseudomonas syringae* DC3000 (He et al 2007).

### OsRap2.6 specifically interacts with RACK1A at WD repeats 1 and 2

We further analyzed the interaction between OsRap2.6 and tryptophan-aspartate (WD) repeats of RACK1A in Y2H assays. RACK1 interacts with co-chaperones, phosphatases and transcription factors through its seven WD (1–7) repeats (Adams et al 2011). We found strong interactions between OsRap2.6 and WD repeats 1 and 2 (Figure 1C). Thus, WD 1 and 2 repeats may be a common binding site for OsRac1 and OsRap2.6 and may possibly act as a potential interaction site or bridge for the three proteins. In another study, when constitutively active OsRac1 (CA-OsRac1) was expressed, it bound RACK1A at WD repeat 1 and 2, enabling OsRac1 to regulate RAR1 and RACK1A at the post-transcriptional levels (Nakashima et al 2008). RACK1 forms homodimers (Liu et al 2007; Thornton et al 2004; Yaka et al 2003) and heterodimers with the remaining WD repeat motifs (3–7) (Chen et al 2004). RACK1 anchors at amino acids 39 and 40 on the 18S ribosomal RNA subunit, constantly mediated by WD repeats 1 and 2 and their associated loops (Adams et al 2011).

### OsRap2.6 localizes in the nucleus and the cytoplasm in rice protoplasts

**Figure 2:**
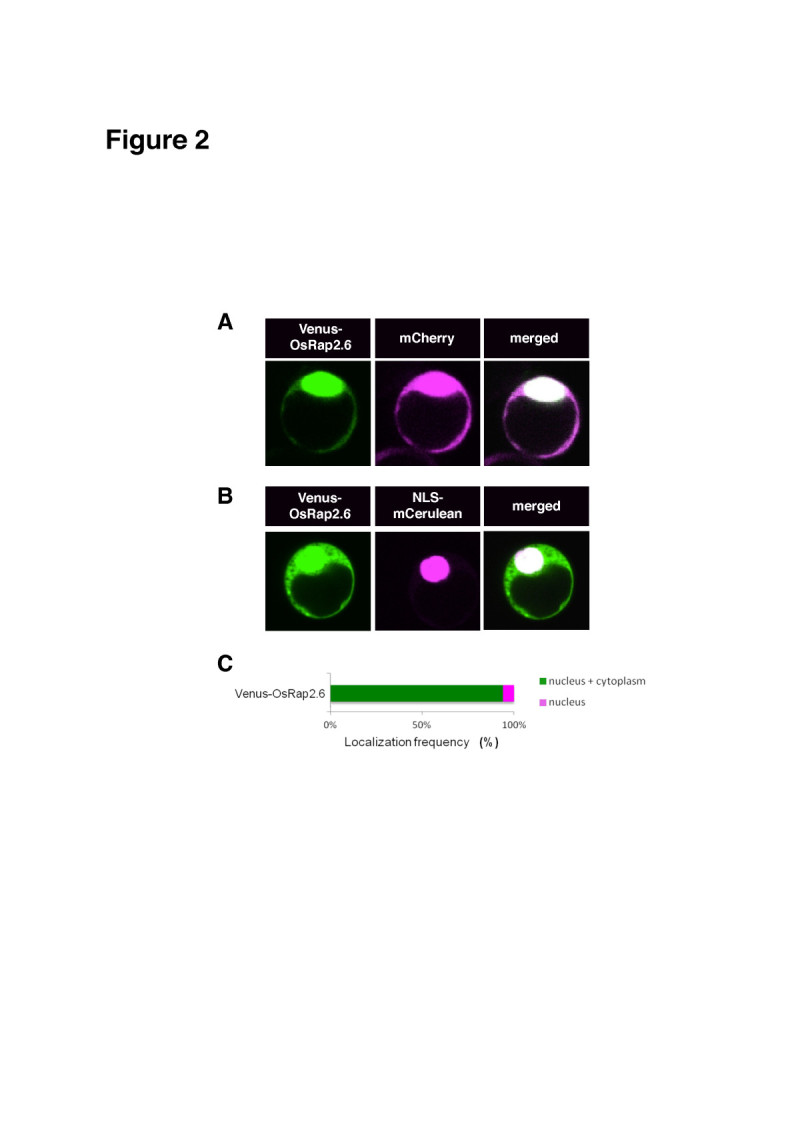
**Subcellular localization of OsRap2.6.** Rice protoplasts were transformed with known fluorescent proteins mCherry and NLS-mCerulean. Fluorescence was detected using a CCD camera connected to a confocal microscope. The localization frequency of the cells was analyzed in 50–100 cells expressing YFP/CFP as compared to the positive controls using Excel. Means and standard deviations were separated using Student’s *t* -test (p<0.05). (**A**) Subcellular localization of Venus-OsRap2.6 with the mCherry. (**B**) Subcellular localization of Venus-OsRap2.6 with the NLS-mCerulean. (**C**) Localization frequency of Venus-OsRap2.6.

To determine the intracellular localization of OsRap2.6 protein, we tagged OsRap2.6 to a variant of yellow fluorescent protein (Venus) at the N-terminus (Venus-OsRap2.6) and expressed the fusion protein in rice protoplasts with the internal positive controls mCherry and mCerulean with a nuclear localization signal (NLS-mCerulean). OsRap2.6 localized with mCherry in the nucleus and the cytoplasm (94%) and the nucleus alone (6%) (Figure 2A). A further comparison using the NLS-mCerulean marker showed 90% of the marker localized within the nucleus and the cytoplasm and 10% in the nucleus alone (Figure 2B). Altogether, OsRap2.6 localized in the nucleus and the cytoplasm in more than 90% of the transformed rice protoplasts.

In Arabidopsis, Rap2.6-YFP and Rap2.6L-YFP localize to the nucleus. In our study, we found a discrepancy between the protein localizations in rice and in Arabidopsis, although both proteins shared a similar NLS. The likely reason for the difference was that Rap2.6 was tagged to the C-terminus, whereas OsRap2.6 was tagged to the N-terminus. Rap2.6 acts as a *trans*-activator in yeast and localizes in the nucleus of onion epidermal cells. A putative nuclear localization signal sequence (RPPKKYRGY), which indicates a possible nuclear localization, is found near the AP2 domain (Zhu et al 2010). A recent report indicates that Rac Immunity 1 (RAI1), a bHLH transcription factor which plays a role in OsRac1-mediated immunity, localizes mainly in the nucleus and partly in the cytoplasm (Kim et al 2012).

### RACK1A localizes in the nucleus and the cytoplasm in rice protoplasts

**Figure 3:**
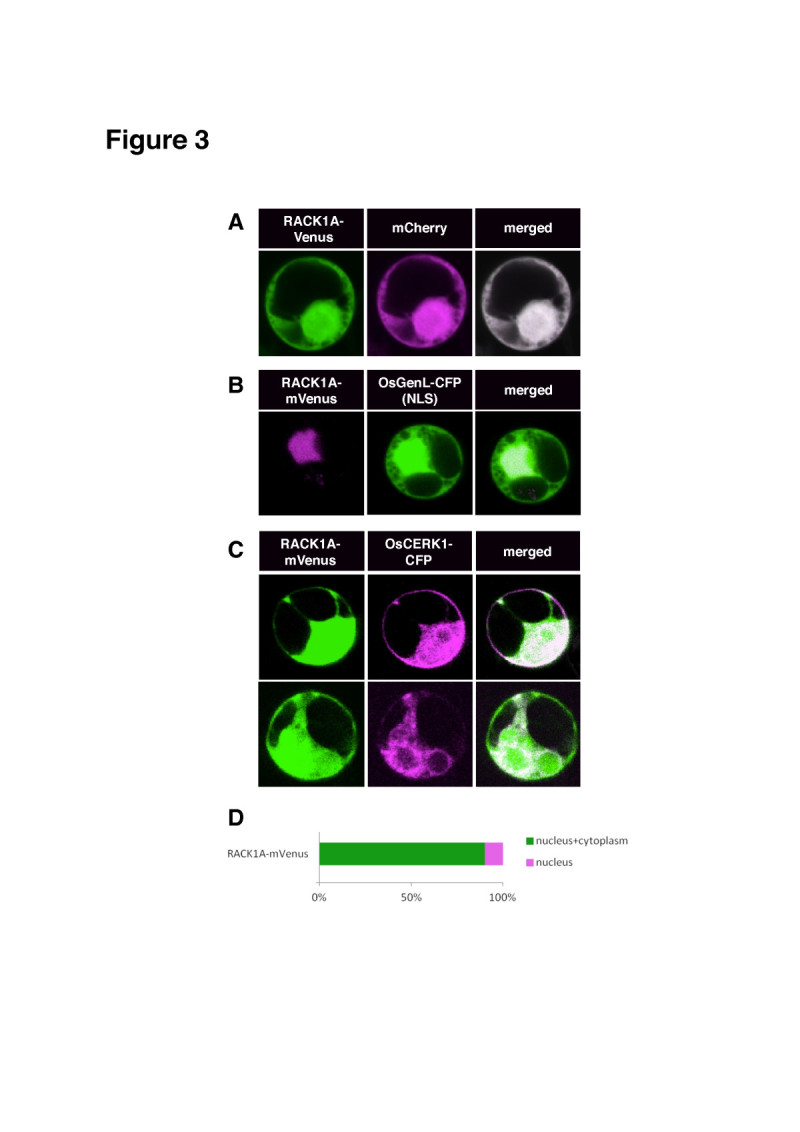
**Subcellular localization of RACK1A in rice protoplasts.** Rice protoplasts were transformed with a plasmid harboring the RACK1A-Venus construct. Protoplasts transformed with known fluorescent proteins mCherry, OsCERK1-GFP and OsGenL-CFP were used as positive controls. Conditions for microscopy and data analysis were identical to those outlined in the legend to Figure 1. (**A**) Subcellular localization of RACK1A-mVenus with mCherry. (**B**) Subcellular localization of RACK1A-Venus with OsGenL-CFP. (**C**) Subcellular localization of RACK1A-Venus with OsCERK1-GFP. (**D**) Localization frequency (%) of RACK1A-Venus.

We expressed RACK1A with the Venus tag at the C-terminus and examined the intracellular localization of this construct with the internal positive controls mCherry, a rice PAMP receptor OsCERK1-GFP (ER and plasma membrane marker) and OsGenL-CFP (nuclear marker). RACK1A-Venus localized mainly in the nucleus and the cytoplasm (CN) (90%) with mCherry (Figure 3A). Moreover, in an independent experiment, RACK1A-Venus co-localized with OsGenL-CFP in the cytoplasm and the nucleus (88%) with the remainder of the CFP localized in the nucleus (12%) (Figure 3B). However, a small proportion (6%) was associated with OsCERK1-GFP at the plasma membrane (PM) and ER (3%) (Figure 3C). Our findings further confirmed the ability of RACK1A to localize as a scaffold protein to different parts of the cell. According to an earlier report, RACK1A localizes in the cytoplasm in rice protoplasts (Nakashima et al 2008). In another report, RACK1A and OsRac1 shifted to detergent-resistant membranes (DRM), regions near PM after elicitation with chitin (Fujiwara et al 2009). Moreover, RACK1A associated with heterotrimeric G-protein’s γ-subunit 2 (RACK1A-AGG2) and localized at PM, the same cellular component where AGG2 is apparently localized; however, RACK1A associated with the γ-subunit 1 (RACK1A-AGG1) throughout the cell (Kamil et al. 2011; Adjobo-Hermans et al 2006). RACK1A modulates its defense responses at posttranscriptional levels through its interaction with OsRac1 in the cytoplasm (Nakashima et al 2008).

### OsRap2.6 and RACK1A interact in the nucleus and the cytoplasm in rice protoplasts

**Figure 4:**
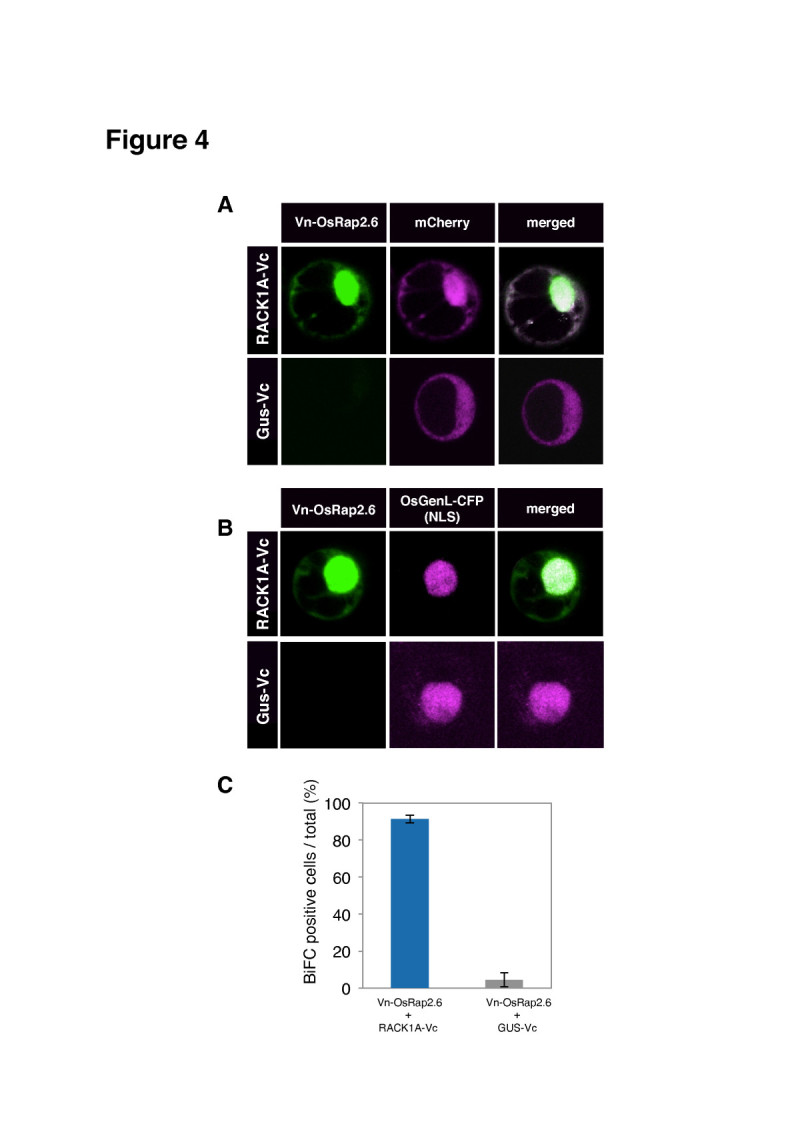
**Interaction of OsRap2.6 with RACK1A in rice protoplasts.** Rice protoplasts were co-transfected with the fluorescent construct (Vn-OsRap2.6 + RACK1A-Vc/GUS-Vc). Conditions for microscopy and data analysis were identical to those outlined in the legend to Figure 1. (**A**) Interaction of OsRap2.6 with RACK1A compared with the mCherry. (**B**) Interaction of OsRap2.6 with RACK1A compared with the OsGenL-CFP. (**C**) Quantitative analysis of BiFC positive cells.

OsRap2.6 and RACK1A localized to the nucleus and the cytoplasm, but the next question was whether the two proteins interact within the same subcellular region or not. We confirmed the *in vivo* interaction using Bimolecular Fluorescence Complementation (BiFC) methods that detect interactions between two proteins in living cells. The absence of an interaction prevents reassembly of the fluorescent protein and results in background fluorescence (Kerppola 2009). We split the Venus fluorescent protein into two halves (Vn/Vc) and tagged OsRap2.6 with Vn and RACK1A with Vc at N and C-termini, respectively. GUS was used as a negative internal control. The paired constructs (Vn-OsRap2.6 + RACK1A-Vc) and (Vn-OsRap2.6 + GUS-Vc) and their controls mCherry and OsGenL-CFP were transfected into rice protoplasts. We found a strong interaction between OsRap2.6 and RACK1A in the cytoplasm and the nucleus and the rest remaining signal in the nucleus with mCherry (Figure 4A). The negative control, GUS, had less than 10% fluorescence in all cells (Figure 4A and C). A further comparison with OsGenL-CFP gave a similar finding (Figure 4B). Our results further confirmed the potential of RACK1A to interact with OsRap2.6 *in vivo*.

As a scaffold protein, RACK1A translocates to different parts of the cell and interacts with different phosphatases and transcription factors (Adams et al 2011). According to a recent report, RACK1A interacts with Arabidopsis Nudix hydrolase (AtNUD7) in the nucleus and the cytoplasm. AtNUD7 expression is induced rapidly in response to an avirulent bacteria and abiotic stresses (Olejnik et al 2011; Kamil et al. 2011). RACK1A forms an interactive complex including OsRac1, RAR1 and SGT1 and maintains an effective conformation, which is able to activate the downstream effectors leading to an immune response (Nakashima et al 2008; Thao et al 2007).

### OsRap2.6 interacts with OsMAPK3 and OsMAPK6 in the nucleus and the cytoplasm

**Figure 5:**
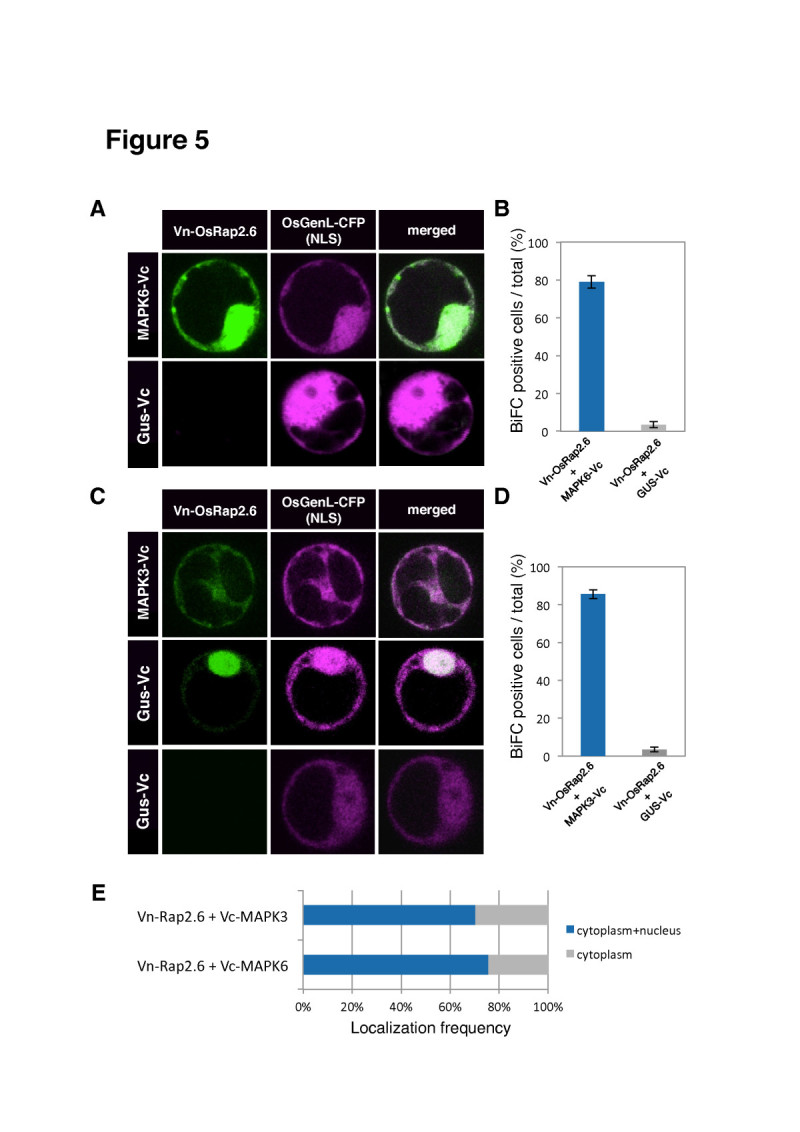
**Interaction of OsRap2.6 with OsMAPK6/3 in rice protoplasts.** Rice protoplasts were co-transfected with the fluorescent constructs (Vn-osRap2.6 + Vc-OsMAPK6) and (Vn-OsRap2.6 + Vc-OsMAPK3) and examined under fluorescence, bright field and overlay. Known fluorescent proteins mCherry, and OsGenL-CFP (nuclear marker) were used as markers of localizations. Conditions for microscopy and data analysis were identical to those outlined in the legend to Figure 1. (**A**) Interaction between OsRap2.6 and OsMAPK6 compared with the mCherry construct. (**B**) Quantitative analysis of BiFC positive cells from (**A**) (OsRap2.6 + OsMAPK6). (**C**) Interaction between OsRap2.6 and OsMAPK3 compared with the mCherry. (**D**) Quantitative analysis of BiFC positive cells from (**C**) (OsRap2.6 + OsMAPK3). (**E**) Frequency (%) of interactions between OsRap2.6 and OsMAPKs in cells.

Mitogen-activated kinase cascades respond to pathogens or pathogen-derived elicitors, for example OsMAPK6 is activated in response to sphingolipid elicitors in rice cell cultures (Kim et al 2012; Lieberherr et al 2005). Furthermore, OsMAPK3 and OsMAPK6 are involved in defense responses in rice (Kim et al 2012; Kishi-Kaboshi et al 2010, Lieberherr et al 2005). We, therefore, investigated whether OsRap2.6 interacts with OsMAPK3 and OsMAPK6. The paired constructs (Vn-OsRap2.6 + Vc-OsMAPK6), (Vn-OsRap2.6 + Vc-OsMAPK3) and their negative control (Vn-OsRap2.6 + GUS-Vc) were transfected into rice protoplasts with mCherry. We found an interaction between OsRap2.6 and OsMAPK6 in the cytoplasm and the nucleus (76%) and (24%) in the cytoplasm. The negative controls had less than 10% fluorescence signal (Figure 5A). In addition, OsRap2.6 and OsMAPK3 interacted in the cytoplasm and the nucleus (72%) and the remainder (28%) of the signal was in the cytoplasm (Figure 5B). Together, these results indicate that OsRap2.6 interacts with OsMAPK6 and OsMAPK3 mainly in the nucleus and the cytoplasm with more than 70% of the cells fluorescencing at the same location.

OsMAPK6 indirectly interacts with CA-OsRac1 in a complex but not with DN-OsRac1 (Lieberherr et al 2005). A complete MAPK cascade (comprised of MEKK1, MKK4/MKK5 and MPK3/MPK6) was proposed to be downstream of the flagellin receptor kinase, FLS2, in Arabidopsis. This signaling cascade activates WRKY22 and WRKY29 transcription factors (Asai et al 2002). Suppression of OsMAPK6 expression by RNAi decreased *PAL1* mRNA levels (Lieberherr et al 2005). RAI1 transcription factor interacts with OsMAPK3 and OsMAPK6 proteins *in vivo* and *in vitro*. Moreover, OsMAPK3/6 and OsMKK4-dd phosphorylate RAI1 *in vitro*. OsBWMK1 is activated in rice leaves after infection with rice blast fungus, elicitor treatment, and wounding (Cheong et al 2003; He et al 1999). OsBWMK1 localizes in the nucleus and phosphorylates OsEREBP1, an ERF transcription factor (Cheong et al 2003). From our findings, we hypothesised that OsRap2.6 may be phosphorylated by OsMAPK3/6 to carry out its transcriptional regulation.

### Chitin elicitor in rice suspension cells induces OsRap2.6 expression

**Figure 6:**
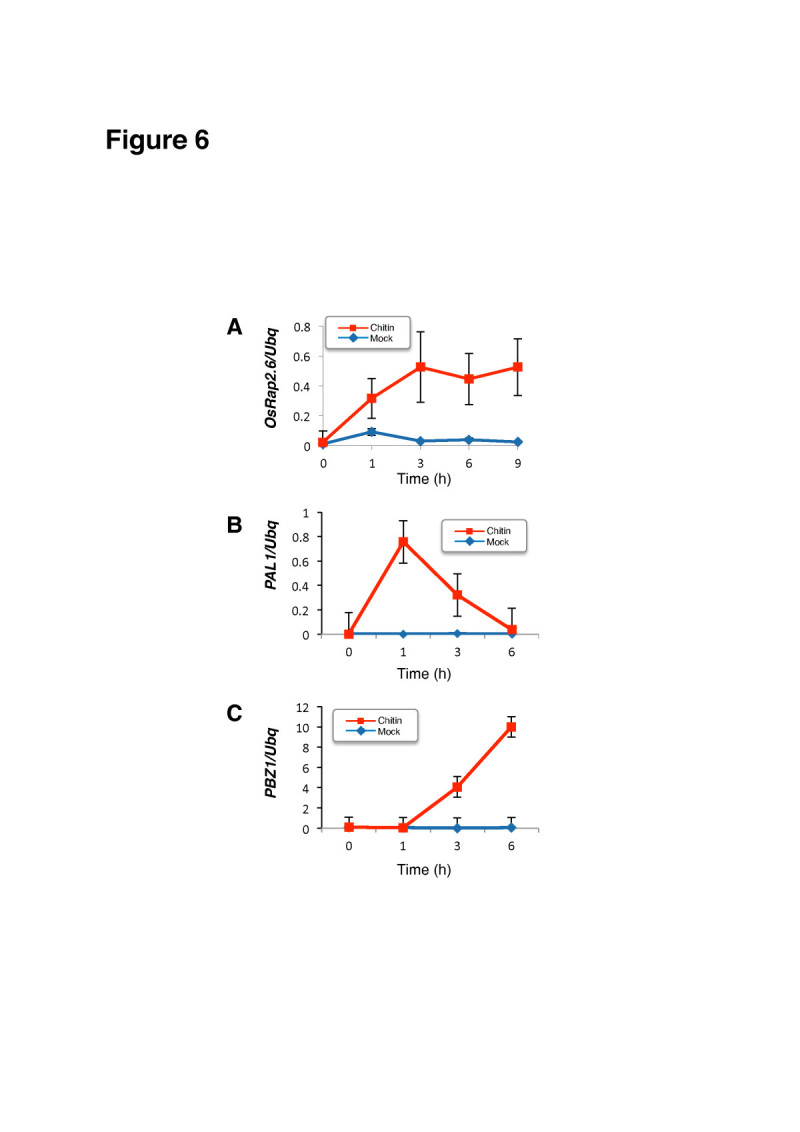
**Induction of** ***OsRap2.6*** **,** ***PAL1*** **, and** ***PBZ1*** **expression induced by chitin in rice suspension cells.** (**A**) *OsRap2.6* expression in WT suspension cells after chitin treatment measured by reverse transcription qPCR. *Ubiquitin* was used as an internal control. (**B**) *PAL1* expression in WT suspension cells after chitin treatment measured by reverse transcription qPCR. (**C**) *PBZ1* expression in WT suspension cells after chitin treatment measured by reverse transcription qPCR.

Suspension cells derived from wild-type japonica cv. Kinmaze rice were treated with chitin (2 μg/ml), and the expression of potential downstream genes, *PAL1, PBZ1* as well as *OsRap2.6* was examined by reverse transcription qPCR. *Ubiquitin* was used as an internal control. *OsRap2.6* transcripts were rapidly increased after chitin treatment and after 3 hr they were not further increased. In contrast to *OsRap2.6*, *PAL* transcripts peaked at 1 hr after chitin treatment and *PBZ1* transcripts started to increase at 3 hr after chitin treatment. (Figure 6A, B and C).

These data agree with recent findings about Rac Immunity1 (RAI1), a bHLH protein, where a gradual increase in *PAL1* and *OsWRKY19* was noted after OsMAPK6 and OsMAPK3 were overexpressed in rice protoplasts (Kim et al 2012). Defense genes *PAL1* and *PBZ1* are rapidly induced by infection with rice blast fungus as previously reported (Chen et al 2010a; Kawano et al 2010; Nakashima et al 2008; Kawasaki et al 1999).

### *OsRap2.6* RNAi plants are susceptible to *M. oryzae* compatible race 007

**Figure 7:**
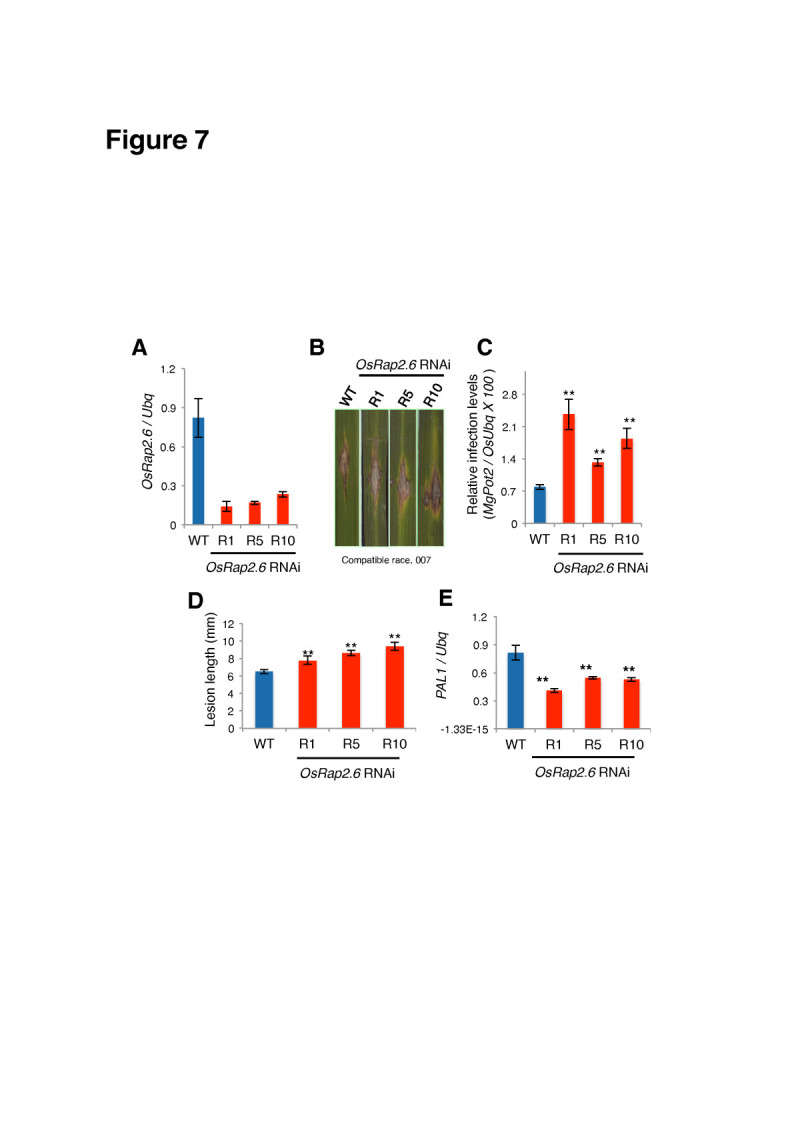
***OsRap2.6*** **RNAi plants are susceptible to a compatible race of** ***M. oryzae.*** *OsRap2.6* transcript levels in RNAi plants were measured by reverse transcription qPCR for three independently transformed lines, R1, R5 and R10. The RNAi plants were grown in the greenhouse for two months and inoculated with *M. oryzae* compatible (virulent) Ina 86–137 (race 007) fungal spore suspensions. (**A**) Expression levels of OsRap2.6 transcripts in T1 *OsRap2.6* RNAi plants before infection with rice blast fungus. (**B**) Photographs showing lesions in leaf blades in WT and *OsRap2.6* RNAi plants. (**C**) Quantitative analysis of fungal growth showing increased susceptibility in *OsRap2.6* RNAi plants 7 days after infection with a compatible race (007) of rice blast fungus. Rice *Ubiquitin* was used as an internal control. Bars represent the means ± SE calculated using four biological replicates where each consists of three independent technical replicates (p ≤ 0.01, n=48). (**D**) Lesion length of *OsRap2.6* RNAi plants compared to WT (p ≤ 0.01, n=48). (**E**) Expression of *PAL1* mRNA in *OsRap2.6* RNAi plants after rice blast infection. Levels of *PAL1* mRNA were down regulated as measured by reverse transcription qPCR (p ≤ 0.01).

We tested if OsRap2.6 contributes to defense responses in rice by constructing *OsRap2.6* RNAi and over-expression (Ox) plants. *OsRap2.6* transcripts from three independent RNAi lines were confirmed by reverse transcription qPCR (R1, R5 and R10) (Figure 7A). The RNAi and Ox plants were grown in the greenhouse for two months and inoculated with *M. oryzae* compatible (virulent) Ina 86–137 (race 007) and incompatible (avirulent) TH67-22 (race 031) fungal spore suspension. *OsRap2.6* RNAi plants showed high susceptibility characterised by larger disease lesions when infected with the compatible race (007) as compared to non-transformed plants (WT) (Figure 7B), qPCR anlysis of fungal growth (p ≤ 0.01, n=48) (Figure 7C) and lesion lengths (p ≤ 0.01, n=48) (Figure 7D). The *PAL1* transcripts were down regulated (p ≤ 0.01) in selected *OsRap2.6* RNAi plants (Figure 7E). Together, these results could indicate that fungal growth was enhanced in *OsRap2.6* RNAi plants as compared to the non-transformed plants. These results demonstrated that OsRap2.6 contributes to defense responses towards compatible rice blast fungus.

We also investigated if *OsRap2.6* RNAi contributes to increased susceptibility to an incompatible blast fungus race (031) in a similar approach as described for the compatible race. From our findings, the susceptibility to the incompatible blast fungus in RNAi plants was not significant as shown in the photograph (Additional file 1: Figure S1A), qPCR analysis of fungal growth (p ≥ 0.05, n= 48) (Additional file 1: Figure S1B) and relative lesion length (p ≥ 0.05, n= 48) (Additional file 1: Figure S1C). Expression of the *PAL1* gene was not significantly reduced in *OsRap2.6* RNAi plants (p ≥ 0.05) (Additional file 1: Figure S1D). Therefore, our results indicate that *OsRap2.6* RNAi does not contribute to increased susceptibility to *M. oryzae* incompatible interactions.

In a study on rice disease resistance, transcription factors including Mybs, WYKYs, NACs and AP2s were induced in leaves infected with blast fungus, indicating the occurrence of transcriptional reprogramming in rice plants after infection (Ribot et al 2008). AP2/EREBPs are also involved in rice viral infections, for example, rice stripe virus (RSV), rice tungro spherical virus (RTSV) and rice dwarf virus (RDV) (Sharoni et al 2011).

*PAL1* is among the 10 most repressed or induced genes in response to *M. oryzae* susceptible interactions (Jantasuriyarat et al 2005). The most highly induced genes in a compatible interaction are *PR-1* and *PR-5* (thaumatin-like proteins), *PBZ1* (*PR-10*), class 11 chitinase (*PR-1a*) and *PAL1* (Kim et al 2012; Chen et al 2010b; Kawano et al 2010; Kim et al 2001). A recent report on RAl1 indicates that *PAL1* and *OsWRKY19* expression increased at 12 and 24 hours in the wild type (control) leaves infected with the compatible rice blast fungus (Kim et al 2012).

### *OsRap2.6* Ox plants have increased resistance to a compatible race of *M. oryzae*

**Figure 8:**
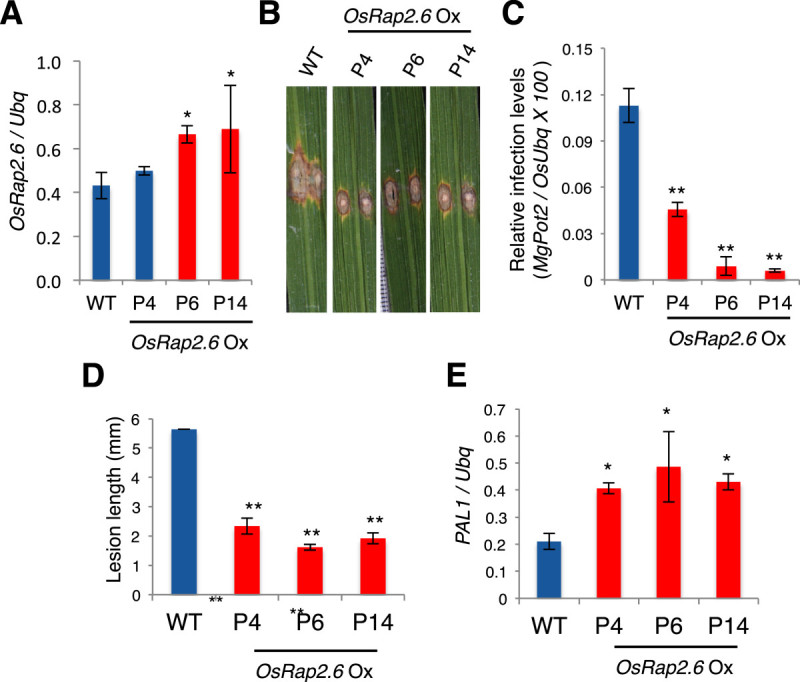
***OsRap2.6*** **Ox plants are resistant to a compatible race (007) of** ***M. oryzae.*** *OsRap2.6* mRNA transcript levels from three independent Ox plants (P1, P6 and P14) were measured by qPCR. The Ox plants were grown in the greenhouse for two months and inoculated with *M. oryzae* compatible (virulent) Ina 86–137 (Race 007) fungal spore suspension. (**A**) Expression levels of OsRap2.6 transcripts in T1 *OsRap2.6* Ox plants before infection with rice blast fungus. (**B**) Photographs showing lesions in leaf blades in WT and *OsRap2.6* Ox plants after infection. (**C**) Quantitative analysis of fungal growth showing increased resistance in *OsRap2.6* Ox plants 7 days after infection with rice blast fungus compatible race (007). *Ubiquitin* was used as an internal control. Bars represent the means ± SE calculated using four biological replicates where each replicate consists of three independent technical replicates (p ≤ 0.01, n=48). (**D**) Lesion length of *OsRap2.6* Ox plants showing increased resistance to blast fungus as compared to WT as shown by increased lesion length after infection (p ≤ 0.01, n=48). (**E**) Expression of *PAL1* mRNA in *OsRap2.6* Ox plants after infection with the compatible race 007. Levels of *PAL1* mRNA were up regulated as measured by reverse transcription qPCR (p ≤ 0.01, n=48).

*OsRap2.6* mRNA transcript levels from three independent Ox plants were measured by reverse transcription qPCR (P4, P6 and P14) (Figure 8A). The plants were infected with the rice blast fungus compatible race, 007. From our findings, smaller disease lesions were observed in *OsRap2.6* Ox plants as compared to the WT as shown in the photograph (Figure 8B), qPCR anlysis of fungal growth (p ≤ 0.01, n= 48) (Figure 8C) and relative lesion length (p ≤ 0.01, n= 48) (Figure 8D). The *PAL1* gene was up regulated (p ≤ 0.01) (Figure 8E). Therefore, *OsRap2.6* Ox showed increased resistance to rice blast fungus compatible interactions.

We also investigated if *OsRap2.6* Ox plants are resistant to the incompatible rice blast fungus race, 031. No significant resistance was noted as shown in the photograph (Additional file 2: Figure S2A), qPCR anlysis of fungal growth (p ≥ 0.05, n= 48) (Additional file 2: Figure S2B), and relative lesion length measurements (p ≥ 0.05, n= 48) (Additional file 2: Figure S2C). Expression of the *PAL1* gene did not significantly increase after infection (p ≥ 0.05) (Additional file 2: Figure S2D). Therefore, *OsRap2.6* Ox does not contribute to disease resistance in incompatible interactions.

These data agreed with other findings; for instance, in response to *M. oryzae*, RACK1A interacted with OsRac1 (Nakashima et al 2008) at the N-terminus of Rboh, leading to ROS production (Wong et al 2007). *RACK1A*-Ox showed increased resistance to a compatible race 007 as compared to the wild type (Nakashima et al 2008). Our findings demonstrated that *OsRap2.6* Ox contributes to increased resistance in compatible interactions.

## Conclusions

Our study confirmed the role of OsRap2.6 in disease resistance to rice blast fungus, and its localization and interaction with RACK1A and OsMAPK3/6 in rice protoplasts. OsRap2.6 possibly localizes in the nucleus when cells are active, during transcriptional regulation, and in the cytoplasm after a stimulus like chitin or a fungus is sensed. OsRap2.6 localization in the nucleus is essential as a transcription factor; furthermore, its interaction with RACK1A is likely to enable it be involved in disease resistance in rice. The interaction with OsMAPK3/6 could potentially lead to phosphorylated OsRap2.6 for transcriptional regulation, a step that is yet to be confirmed. We found OsRap2.6 to be a positive regulator in *M. oryzae* compatible interactions possibly as a downstream signal of RACK1A. This study has opened up other areas for further research such as the analysis of OsRap2.6 target genes in the defense response pathway.

## Methods

### Comparison of predicted amino acid sequences of OsRap2.6

To identify OsRap2.6-related genes, the sequence of OsRap2.6 was used as a query for BLAST searches in the rice and Arabidopsis genome databases (http://www.ncbi.nlm.nih.gov/nuccore). Highly similar amino acid sequences were aligned with the OsRap2.6 sequence using Genetyx software for Mac-Pro, Version 10 (Genetyx, USA).

### Yeast two-hybrid assays

The bait constructs, RACK1A, OsRac1 (WT) and (CA and DN) coding regions were ligated to the pBTM116 vector, and OsRap2.6 was ligated to the prey vector, pVP16 as described previously (Nakashima et al 2008; Kawasaki et al 1999). The negative controls were pBTM116ss and pVP16. The vectors concentrations ranged between 150–200 ng/μl hosted by the yeast *Saccharomyces cerevisiae* L40 (25 μl). The cells were cultured on synthetic complete medium lacking uracil and tryptophan, either with histidine (SC-UW) or without histidine (SC-UWLH). The inhibitor 3-amino-1, 2, 4-triazole (3-AT) (3 mM), was included in the SC-UWLH media.

### OsRap2.6, RACK1A and OsMAPK3/6 constructs

An entry clone, pENTR-OsRap2.6 was amplified from pVP16-OsRap2.6 (0.5 μl) with forward (5’-CACCATGGTCACCGCGCTAGCCACGT-3’) and reverse (5’-TCACGACGACGAATCCTTCTTCTTG-3’) primers. The blunt-end PCR product was cloned into pENTR-D/TOPO as per the manufacturer’s instructions (Invitrogen, USA). Colonies were selected with M13 forward (5’-TGTAAAACGACGGCCAGT-3’) and reverse (5’-CAGGAAACAGCTATGAC-3’) primers. The pENTR-OsRap2.6 was ligated into Gateway destination vectors (GW) with the LR clonase II enzyme (0.5 μl) (Invitrogen, USA) whose expression was driven by the 35S-Cauliflower mosaic virus promoter (35S-Vn-OsRap2.6).

For the subcellular localization studies, we used BiFC in which Venus, a variant of YFP, was split into the N-terminal and C-terminal halves (Vn/Vc). The N-terminal half of Venus (Vn) was tagged to OsRap2.6 (Vn-OsRap2.6) and sequenced with pB12221-35S forward primer and the *nos* terminator as the reverse primer as listed in Table 2. RACK1A-Venus constructs were provided from our laboratory stocks. The half Venus constructs (Vc-OsMAPK3) and (Vc-OsMAPK6) were described previously (Kim et al 2012). The DNA sequence of all plasmids was confirmed in using an ABI-PRISM Big Dye Terminator Cycle Sequencing Kit with an ABI PRISM 310 Genetic Analyzer (Applied Biosystems, USA). Data were analysed using Genetyx software for the Mac-Pro, Version 10 (Genetyx, USA).

**Table 2:** **Primers used to sequence RNAi and Ox constructs**

Primer	Sequence
Ubq 1st intron forward	5’-GCTCTAACCTTGAGTACCTATCTA-3’
Ubq 1st intron forward	5’-TTATCGCATACTTCCGTCCCGAT-3’
Nos terminator reverse	5’-CCATCTCATAAATAACGTCATGCAT-3’
Nos terminator reverse	5’- AGACAACTTAATGCAATTCGTACAT-3’
GUS linker forward	5’-CGTCGGTGAACAGGTATGGAATT-3’
GUS linker forward	5’-TTATACGGAACGCTCCAGCGTT-3’
GUS linker reverse	5’-CACGTAAGTCCGCATCTTCATGA-3’
GUS linker reverse	5’-CGCTTGTCAAGGACTAATTGGTG-3’
*attB1* forward	5’-AGTTTGTACAAAAAAGCAGGCTCC-3’
*attB2* reverse	5’-GCTGGGTCGAAAGAACATGTTTCA-3’
Real time *PAL* forward	5’-TGAATAACAGTGGAGTGTGGAG-3’
Real time *PAL* reverse	5’-AACCTGCCACTCGTACCAAG-3’
Real time *PBZ1* forward	5’-ATGAAGCTTAACCCTGCCGC-3’
Real time *PBZ1* reverse	5’-GTCTCCGTCGAGTGTGACTTG-3’
Real time *ubiquitin* forward	5’-AACCAGCTGAGGCCCAAGA-3’
Real time *ubiquitin* reverse	5’-ACGATTGATTTAACCAGTCCATGA-3’
*M. grisea Pot2* forward	5’-ACGACCCGTCTTTACTTATTTGG-3’
*M. grisea Pot2* reverse	5’-AAGTAGCGTTGGTTTTGTTGGAT-3’
*OsRap2.6* forward	5’-CACCCGGCACCTGGACAGAACAGATCA-3’
*OsRap2.6* reverse	5’ -AGAATCCTCTCTCTTGCTTTACTTGGAC-3’
*Rap2.6* forward	5’-GAGCCTGACCTATTGCATCTCC-3’
*Rap2.6* reverse	5’-GGCCTCCAGAAGAAGATGTTGG-3’
pB12221-35S forward	5’-ACTGACGTAAGGATGACGC-3’
*nos* terminator	5’-GATAATCATCGCAAGACCG-3’
*OsRap2.6* Ox forward	5’-CACCATGGTCACCGCGCTAGCCCACGTCA-3’
*OsRap2.6* Ox reverse	5’-GAACGATCGGGGAAATTCGAGCTC-3’

Primer names and oligonucleotide sequences used for sequencing.

### Isolation of rice protoplasts, transfection and BiFC

For effective protoplast isolation, suspension cells were crushed from primary calli into small pieces prior to enzyme treatment. The protoplasts were adjusted to a density of 1.5-2 x 10^7^ cells/ml (Kyozuka et al 1987). For intracellular localization studies, 100 μl of protoplasts were transfected with 9–10 μg plasmids (Venus-OsRap2.6) or (RACK1A-Venus) and/or control plasmids Cherry, NLS-Cerulean and OsGenL-CFP (nuclear marker). The BiFC system used in this study was as described previously with slight modifications (Chen et al 2010a; Kawano et al 2010; Kakita et al. 2007). For the interaction studies, protoplasts (100 μl) (1.5-2 × 10^6^ cells) were transformed with 2.5-5 μg of each paired construct (Vn-OsRap2.6 + RACK1A-Vc), (Vn-OsRap2.6 + Vc-OsMAPK3/6) and a negative control (Vn-OsRap2.6 + GUS-Vc) by the polyethylene glycol (PEG) method with minor modifications (Yoo et al 2007). The protoplasts were incubated at 30°C for 15 hours. The localization or co-localization of YFP/CFP proteins and their markers was assessed with a confocal microscope (Leica TCS SP5) in sequential scanning mode as described in the next section. Quantitative assays were accomplished using a method described previously where 50–100 cells of each construct were randomly scanned and categorized according to their plasma membrane (PM), cytoplasm (C), nuclear (N), or cytoplasm and nuclear (CN) localization patterns.

### Confocal scanning microscopy

Confocal scanning microscope was used to image the rice protoplasts expressing fluorescent proteins. The microscope was equipped with the Leica confocal software (LCS), a 100mW multi-line Argon laser (458nm, 476nm, 488nm, 496nm and 514nm), diode pumped solid state laser (DPSS) (442nm), a 10mW DPSS (561nm), a 10mW He-Ne Laser (633nm) and a 50mW UV laser (351nm-364nm) as excitation sources. The SP scanner collected the FP signal at various wavelengths, and the auto fluorescence of the protoplasts was measured between 440 nm and 650 nm. LCS carried out the image maximal projection. Images were acquired using the 10x/0.4 HC PLAPO CS object lens and the 40x/0.85 HCX PLAPO CS object lens. The 63x/1.2 HCX PLAPO CS and 40x 1.25-0.70 HCX PL APO CS object lenses were used to obtain images when fluorescent proteins were targeted to any location.

### RNAi, Ox constructs and rice transformation

To generate an RNAi construct for gene suppression, a 300 base pair fragment was amplified by PCR from OsRap2.6 with the *OsRap2.6* RNAi primers listed in Table 2. The open reading frame (ORF) of the *OsRap2.6* construct was amplified using the *OsRap2.6* Ox primers listed in Table 2. The PCR fragments were cloned into the Gateway pENTR/D-TOPO cloning vector. Subsequently, the derived fragments were transferred to the pANDA destination vector by recombinase (LR) reactions. The pANDA vector has kanamycin and hygromycin resistance markers for transformation (Miki and Shimamoto 2004). The insert and vector sequences were confirmed by PCR using the first intron of *Ubiquitin*, the *nos* terminator, GUS linker and *attribute* B1 and B2 primers listed in Table 2.

### Suspension cells, RNAi and over-expressing plants

*OsRap2.6* RNAi and Ox calli were derived from japonica rice cv. Kimnaze. The seeds were surface sterilised with 1.2% sodium hypochlorite for 45 min, washed in distilled water and placed on Murashige and Skoog (MS) medium supplemented with 2 mg/L, 2, 4-dichlorophenoxyacetic acid (2,4-D) (Murashige and Skoog 1962). Plants were generated by *Agrobacterium tumifaciens*-mediated transformation of rice callus as described previously (Miki and Shimamoto 2004; Hiei et al 1994). The transformed callus was selected with forward (5’-TGGCGGCTACTACCCCTCGTCGT-3’) and reverse (5’-GAACGATCGGGGAAATTCGAGCTC-3’) primers. The suspension culture derived from transformed callus was maintained in R2S medium (Ohira et al 1973). OsRap2.6 RNAi plants were screened using PCR with *Rap2.6* primers listed in Table 2.

### RNA extraction and reverse transcription PCR

For the analysis of gene expression, rice calli from the WT suspension cells were treated with 2 μg/ml chitin (Hepta-N-acetylchitoheptaose, Sigma) and harvested at different time intervals (Lieberherr et al 2005). The samples were frozen in liquid nitrogen and stored at –80°C. Briefly, RNA was extracted by the TRIzol method (Nacalai tesque, Japan). The samples were digested with DNaseI (Takara, Shiga, Japan). Electrophoresis was done in 1.5% agarose gels in 1 X TBE buffer, at 100V for 30 min. The gels were stained with ethidium bromide for 15 min. Bands were visualized under UV light.

### Infection of rice plants with *M. oryzae*

*OsRap2.6* RNAi and Ox plants were infected with the compatible race (007) or the incompatible race (031) of rice blast fungus (*M. oryzae*). The fungal growth conditions and the punch infection method were done as described previously with minor modifications (Kim et al 2012; Chen et al 2010a; Kawano et al 2010). The spores were estimated to contain ~ 1 × 10^5^ spores per ml. The spore suspension was inoculated on leaf blades and kept at 23~30°C in the greenhouse. Disease lesions sizes were measured 7 days after inoculation. Briefly, the two youngest leaf blades were selected for infection. Six holes were punched per blade in 4 plants giving a total of 48 infected sampling points. Lesion length was measured quantitatively with a digital calliper. The resistance and susceptibility of each plant was compared with the wild type using four cv. Kinmaze plants. The experiment was repeated three times. The data were analysed for statistical significance using the Excel program (Microsoft). The means and standard errors were analysed and the p-values were determined by a standard *t* -test (p<0.05).

### Quantitative PCR (qPCR)

We used the standard curve quantification method that absolute values were derived from known quantities. The qPCR mixture (20 μl) was loaded into Ultra AMP PCR plates and analysed in an ABI StepOne Real-Time PCR System for 2.5 hours. To detect *M. grisea* and rice DNAs, two sets of primers against *M. grisea Pot2* and rice *Ubiquitin* were used in qPCR (Beruyer et al 2006)*.* The DNA representing the relative number of fungus cells was quantified per plant cell from the infected rice tissues by calculating an infection ratio with the formula (N: Mgpot2/ N: *Osubiquitin* x 100) as described previously (Kawano et al 2010). DNA was extracted from the infected lesions and analysed further qualitatively in qPCR (ABI StepOne Real-Time PCR System) using *M. grisea Pot2*, *PAL1*, *PBZ1* and *Ubiquitin* primers listed in Table 2.

## Electronic supplementary material

Additional file 1:**Figure S1.** *OsRap2.6* RNAi plants are not susceptible to *M. oryzae,* incompatible race, 031. (A) Fungal infections on leaf blades of WT and *OsRap2.6* RNAi after infection with 031, an incompatible race of rice blast fungus. R1, R5 and R10 are independently transformed lines. (B) Quantitative analysis of fungal growth in *OsRap2.6* RNAi, 7 days after infection. *Ubiquitin* was used as an internal control. Bars represent the means ± SD calculated using four biological replicates where each consists of three independent technical replicates (p ≥ 0.05, n=48). (C) Lesion length of *OsRap2.6* RNAi plants showing susceptibility to blast fungus incompatible race, 031 as compared to WT (p ≥ 0.05, n=48). (D) Expression of *PAL1* mRNA in *OsRap2.6* RNAi after rice blast infection with the incompatible race, 031. Levels of *PAL1* mRNA were measured by reverse transcription qPCR (p ≥ 0.05, n=48). (PDF 240 KB)

Additional file 2:**Figure S2.** *OsRap2.6* Ox plants were not resistant to *M. oryzae,* incompatible race, 031. (A) Fungal infections on leaf blades of WT and *OsRap2.6* Ox plants in incompatible race, 031. P4, P6, and P14 are independently transformed lines. (B) Quantitative analysis of fungal growth in *OsRap2.6* Ox plants 7 days after infection with the incompatible race (031) of rice blast fungus. *Ubiquitin* was used as an internal control. Bars represent the means ± SD calculated using four biological replicates where each consists of three independent technical replicates (p ≥ 0.05, n=48). (C) Lesion length of *OsRap2.6* Ox showing susceptibility to blast fungus incompatible race, 031 as compared to WT. (D) Expression of *PAL1* mRNA in *OsRap2.6* Ox plants after infection (p ≥ 0.05). (PDF 2 MB)

Below are the links to the authors’ original submitted files for images.

Authors’ original file for figure 1

Authors’ original file for figure 2

Authors’ original file for figure 3

Authors’ original file for figure 4

Authors’ original file for figure 5

Authors’ original file for figure 6

Authors’ original file for figure 7

Authors’ original file for figure 8

## Abbreviations

AP2: Apetala2
ERF: Ethylene-responsive element-binding proteins
bHLH: Basic Helix Loop Helix
BiFC: Bimolecular fluorescence complementation
CA-OsRac1: Constitutively active-OsRac1
CCR1: Cinnamoyl CoA reductase 1
CC-NB-LRR: Coiled-coil NB-LRR
CERK1: a LysM receptor kinase essential for chitin elicitor signaling
CFP: Cyan fluorescent protein
DN-OsRac1: Dominant negative-OsRac1
DREB: Dehydration-responsive element-binding protein
DRMs: Detergent resistant membranes
ER: Endoplasmic reticulum
ERF: Ethylene-responsive factor
ETI: Effector triggered immunity
FLS2: Flagellin sensing 2
GAP: GTPase-activating protein
GTP: Guanosine triphosphate
GDP: Guanosine diphosphate
GEF: Guanine exchange factors
GFP: Green fluorescent protein
HR: Hypersensitive responses
Hsp70: Heat shock protein 70
Hsp90: Heat shock protein 90
LCM: Leica confocal software
MAPK 3/6: Mitogen-activated protein kinases 3 and 6
NADPH: Nicotinamide adenine dinucleotide phosphate
NB-LRR: (NB)-leucine rich repeat (LRR)
NB-ARC: ARC: APAF-1, certain R gene products and CED-4
NLS: Nuclear localisation signal
NO: Nitric oxide
OsRac1: Rice small GTPase, Rac1
OsRACK1: A rice Receptor for Activated Kinase C1 (RACK1)
OsRap2.6: A rice transcription factor, Rap2.6
Ox: Overexpressing
PAL1: Phenylalanine ammonia-lyase 1
PBZ1: Probenazole-induced protein 1
PAMP: Pathogen associated molecular pattern
PKC: Protein kinase C
PM: Plasma membrane
PR genes: Pathogen related genes
qPCR: Real time polymerase chain reaction
R protein: Resistance protein
RAl1: Rac immunity 1
RACK1: Receptor for activated Kinase C-1
RDV: Rice dwarf virus
RLK: Receptor-like kinases
ROS: Reactive oxygen species
RSV: Rice stripe virus
RTSV: Rice tungro spherical virus
SA: Salicyclic acid
TIR: Toll/ interleukin-1 receptor (TIR) or
Vn/Vc: Venus N terminus and C terminus
YFP: Yellow fluorescent protein
Y2H: Yeast two-hybrid.
