# Supplementary figures and images for: OsRap2.6 transcription factor contributes to rice innate immunity through its interaction with Receptor for Activated Kinase-C 1 (RACK1)
Source: Rice (N Y). 2012 Dec 11;5:35. doi: 10.1186/1939-8433-5-35 (PMC4883712; doi:10.1186/1939-8433-5-35)

# Supplementary Figure S1

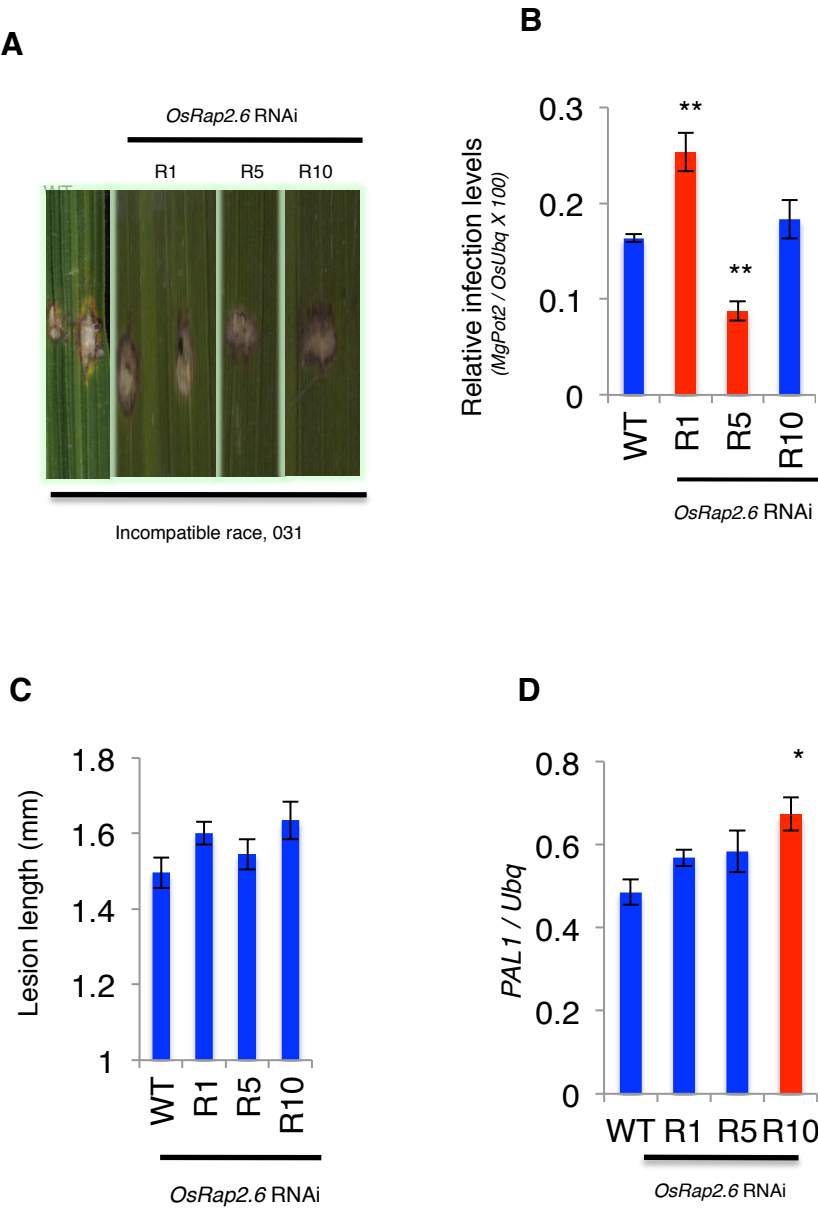

Supplement: Supplementary file 1 — Additional file 1:Figure S1. OsRap2.6 RNAi plants are not susceptible to M. oryzae, incompatible race, 031. (A) Fungal infections on leaf blades of WT and OsRap2.6 RNAi after infection with 031, an incompatible race of rice blast fungus. R1, R5 and R10 are independently transformed lines. (B) Quantitative analysis of fungal growth in OsRap2.6 RNAi, 7 days after infection. Ubiquitin was used as an internal control. Bars represent the means ± SD calculated using four biological replicates where each consists of three independent technical replicates (p ≥ 0.05, n=48). (C) Lesion length of OsRap2.6 RNAi plants showing susceptibility to blast fungus incompatible race, 031 as compared to WT (p ≥ 0.05, n=48). (D) Expression of PAL1 mRNA in OsRap2.6 RNAi after rice blast infection with the incompatible race, 031. Levels of PAL1 mRNA were measured by reverse transcription qPCR (p ≥ 0.05, n=48). (PDF 240 KB) [file 12284_2012_33_MOESM1_ESM.pdf]

# Supplementary Figure S2

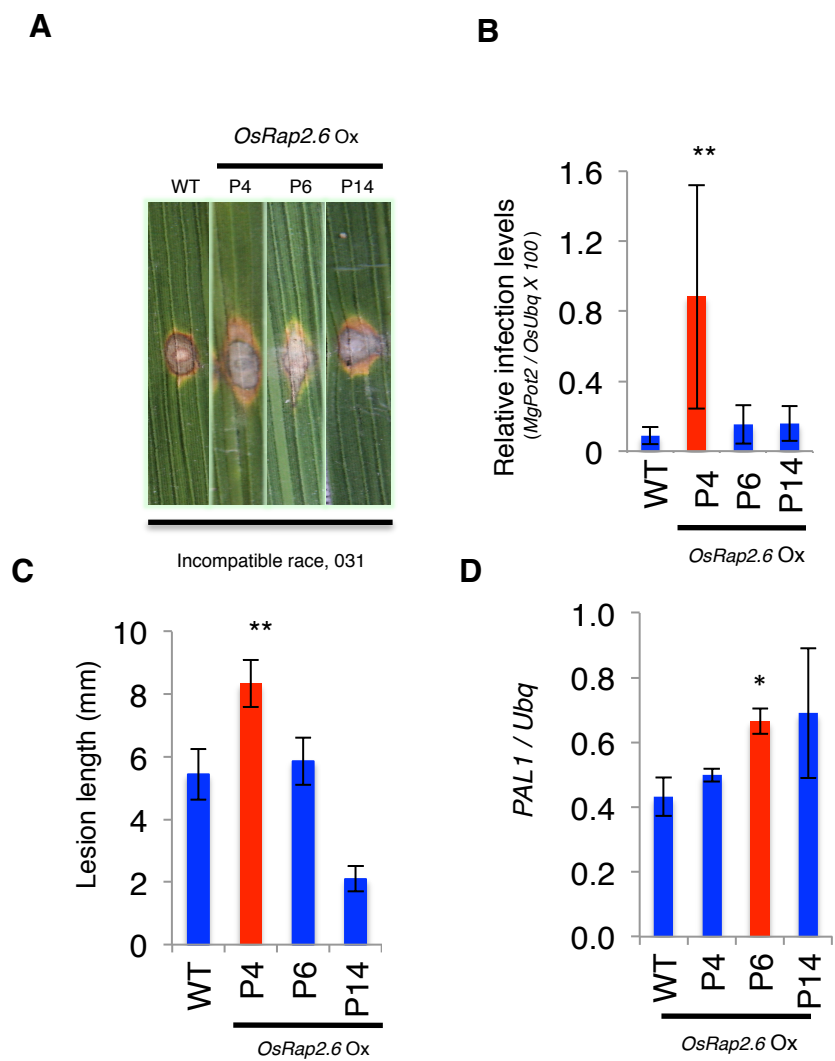

Supplement: Supplementary file 2 — Additional file 2:Figure S2. OsRap2.6 Ox plants were not resistant to M. oryzae, incompatible race, 031. (A) Fungal infections on leaf blades of WT and OsRap2.6 Ox plants in incompatible race, 031. P4, P6, and P14 are independently transformed lines. (B) Quantitative analysis of fungal growth in OsRap2.6 Ox plants 7 days after infection with the incompatible race (031) of rice blast fungus. Ubiquitin was used as an internal control. Bars represent the means ± SD calculated using four biological replicates where each consists of three independent technical replicates (p ≥ 0.05, n=48). (C) Lesion length of OsRap2.6 Ox showing susceptibility to blast fungus incompatible race, 031 as compared to WT. (D) Expression of PAL1 mRNA in OsRap2.6 Ox plants after infection (p ≥ 0.05). (PDF 2 MB) [file 12284_2012_33_MOESM2_ESM.pdf]

**A**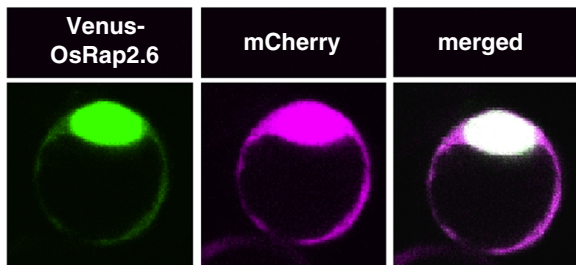**B**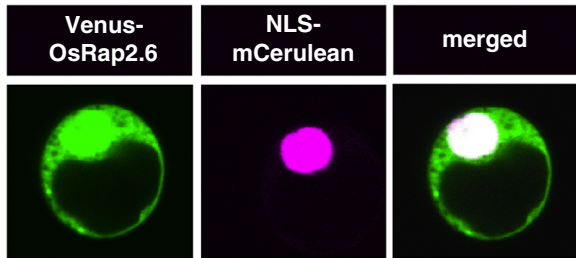**C**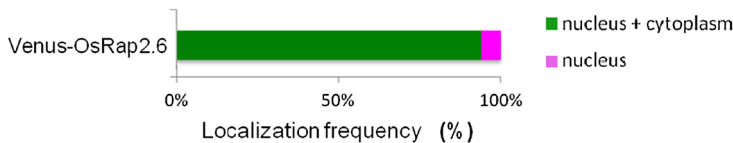

Supplement: Supplementary file 4 — Authors’ original file for figure 2 [file 12284_2012_33_MOESM4_ESM.pdf]

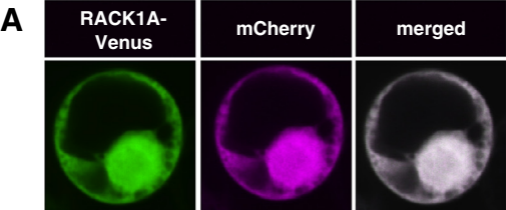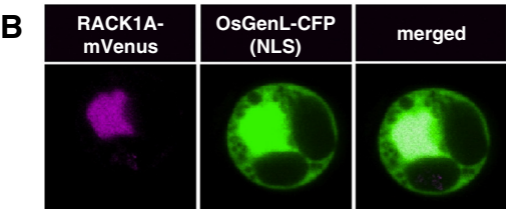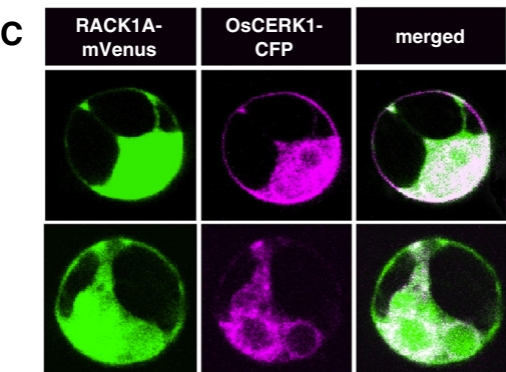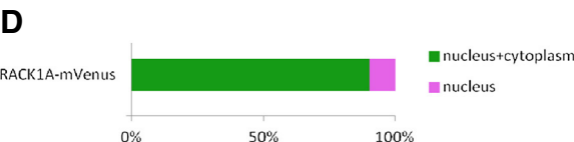

Supplement: Supplementary file 5 — Authors’ original file for figure 3 [file 12284_2012_33_MOESM5_ESM.pdf]

**A**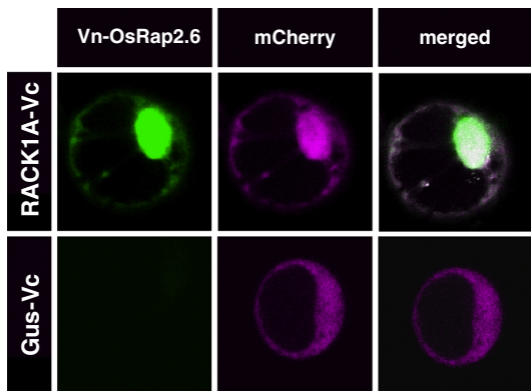**B**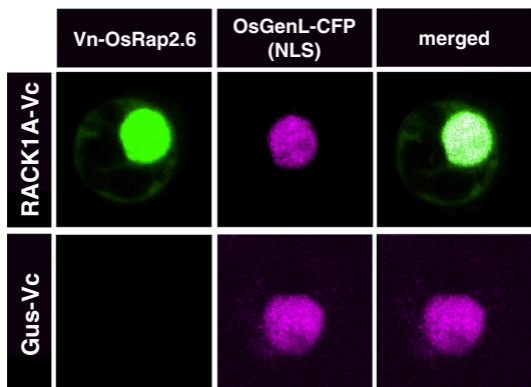**C**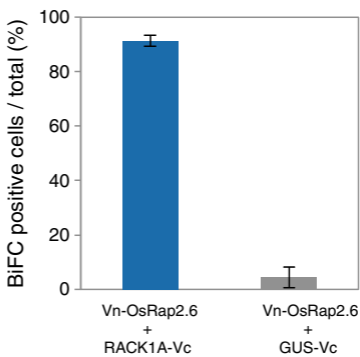

Supplement: Supplementary file 6 — Authors’ original file for figure 4 [file 12284_2012_33_MOESM6_ESM.pdf]

**A**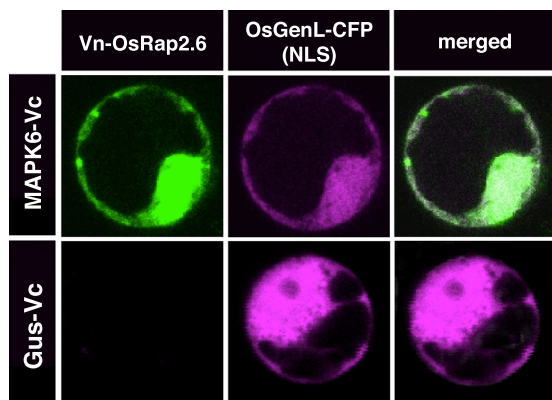**B**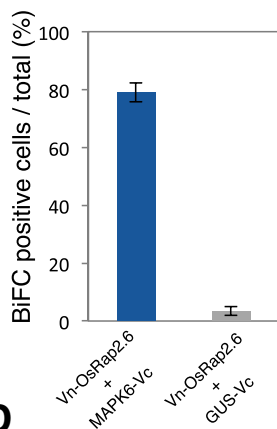**C**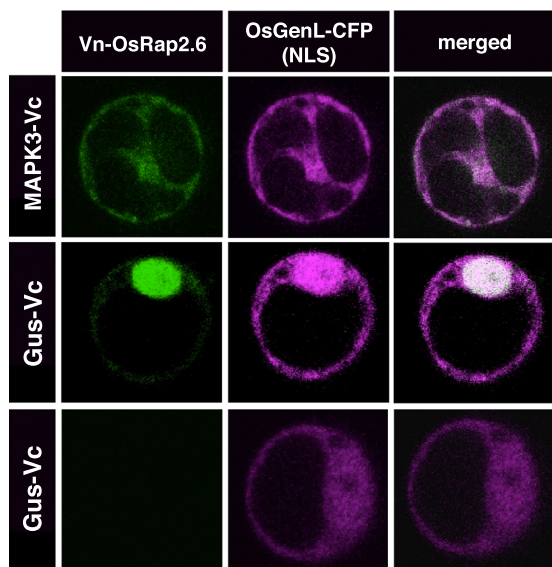**D**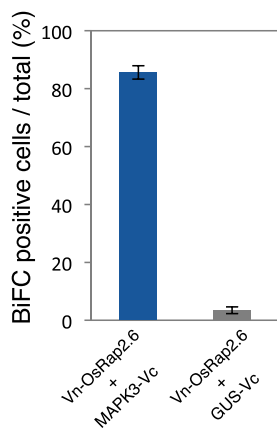**E**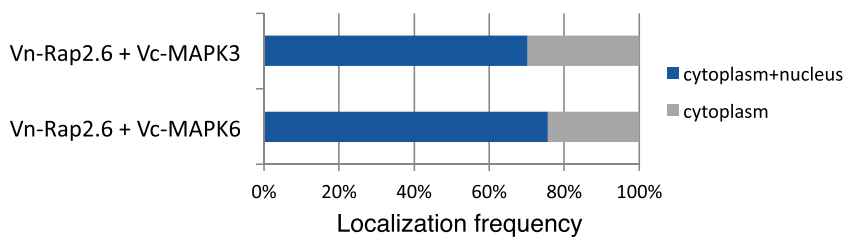

Supplement: Supplementary file 7 — Authors’ original file for figure 5 [file 12284_2012_33_MOESM7_ESM.pdf]

**A**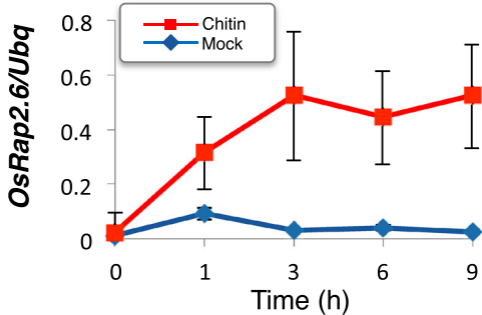**B**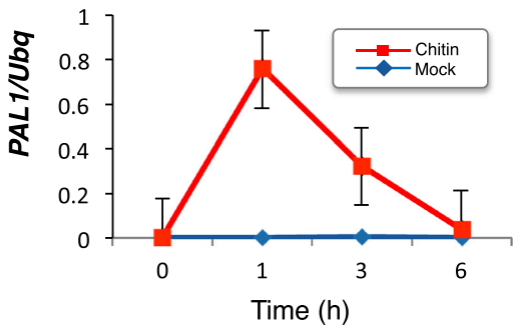**C**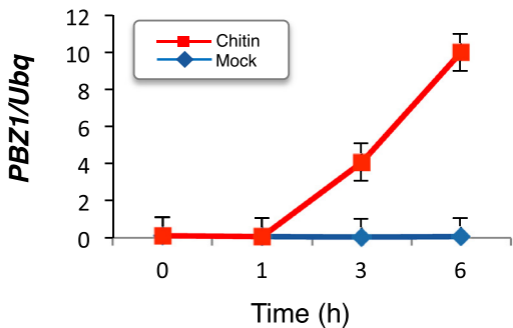

Supplement: Supplementary file 8 — Authors’ original file for figure 6 [file 12284_2012_33_MOESM8_ESM.pdf]

**A**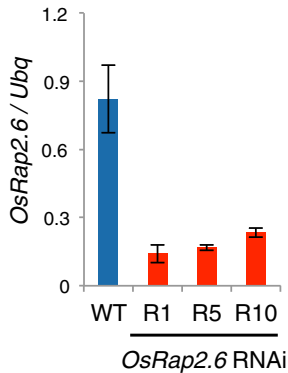**B**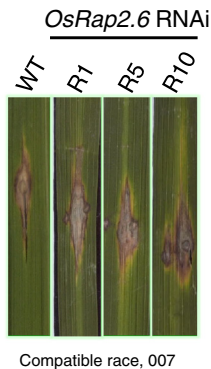**C**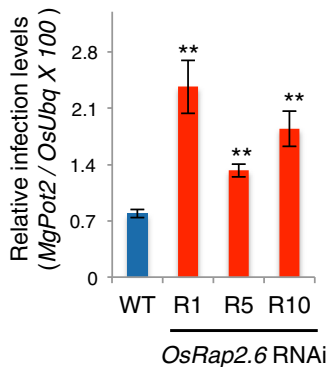**D**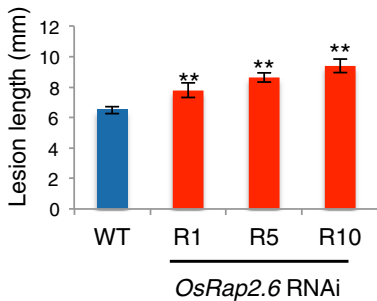**E**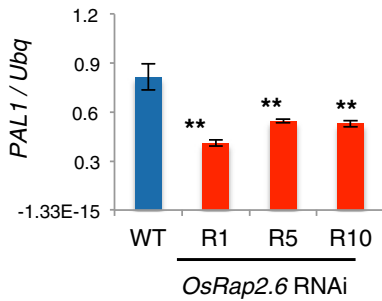

Supplement: Supplementary file 9 — Authors’ original file for figure 7 [file 12284_2012_33_MOESM9_ESM.pdf]

**A**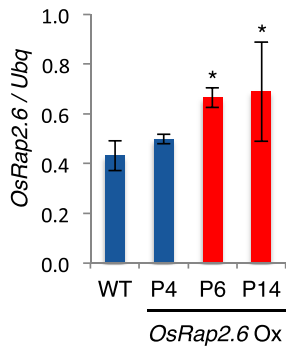**B**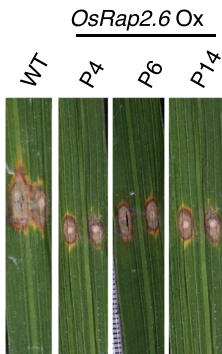**C**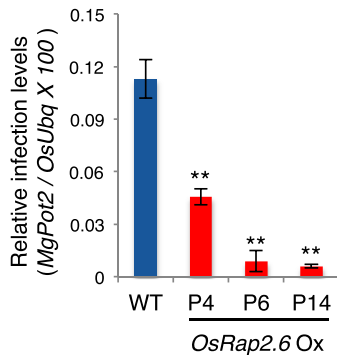**D**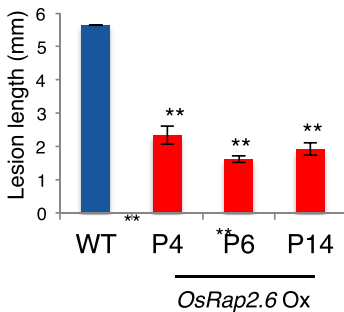**E**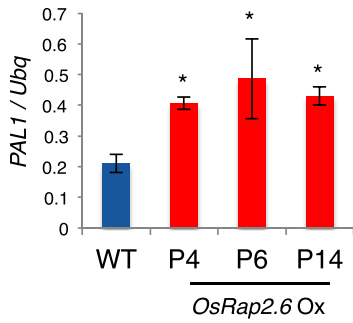

Supplement: Supplementary file 10 — Authors’ original file for figure 8 [file 12284_2012_33_MOESM10_ESM.pdf]
