# Supplementary material for: OsRap2.6 transcription factor contributes to rice innate immunity through its interaction with Receptor for Activated Kinase-C 1 (RACK1)
Source: Rice (N Y). 2012 Dec 11;5:35. doi: 10.1186/1939-8433-5-35 (PMC4883712; doi:10.1186/1939-8433-5-35)
Supplement: Supplementary file 3 — Authors’ original file for figure 1 [file 12284_2012_33_MOESM3_ESM.pdf]

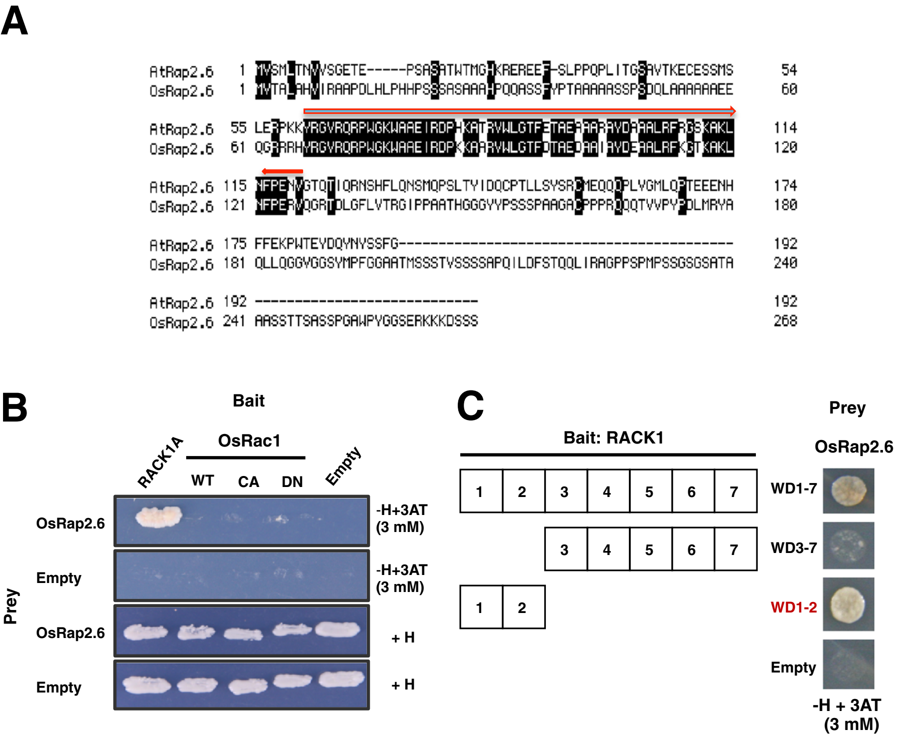

A
AtRap2.6 1 MVSMLTNVVSGETE-----PSASATWTMGHKREREEF-SLPPQPLITGSAVTKECESSMS 54
OsRap2.6 1 MVTALAHVIRAAPDLHLPHHPSSSASAAAHPQQASSFYPTAAAAASSPSDQLAAAAAAEE 60
AtRap2.6 55 LERPKKVRGVRQRPWGKWAAEIRDPHKATRVWLGTFETAEAAARAYDAAALRFRGSKAKL 114
OsRap2.6 61 QGRRRHVRGVRQRPWGKWAAEIRDPKKAARVWLGTFDTAEDAAIAYDEAALRFKGTKAKL 120
AtRap2.6 115 NFPENVGTQTIQRNSHFLQNSMQPSLTYIDQCPTLLSYSRCMEQQDPLVGMLQPTEEENH 174
OsRap2.6 121 NFPERVQGRTDLGFLVTRGIPPAATHGGGYYPSSSPAAGACPPPRDQQTVVPYPDLMRYA 180
AtRap2.6 175 FFEKPWTEYDQYNYSSFG-- 192
OsRap2.6 181 QLLQGGVGGSYMPFGGAATMSSSTVSSSSAPQILDFSTQQLIRAGPPSPMPSSGSGSATA 240
AtRap2.6 192 ---------------------------- 192
OsRap2.6 241 AASSTTSASSPGAWPYGGSERKKKDSSS 268
B
Bait
OsRac1
RACK1A
WT
CA
DN
Empty
Prey
OsRap2.6
Empty
OsRap2.6
Empty
-H+3AT (3 mM)
-H+3AT (3 mM)
+ H
+ H
C
Bait: RACK1
1 2 3 4 5 6 7
3 4 5 6 7
1 2
Prey
OsRap2.6
WD1-7
WD3-7
WD1-2
Empty
-H + 3AT (3 mM)
